# Genetic Predisposition to Primary Lactose Intolerance Does Not Influence Dairy Intake and Health-Related Quality of Life in Romanian Children: A Hospital-Based Cross-Sectional Study

**DOI:** 10.3390/children10061075

**Published:** 2023-06-18

**Authors:** Corina Pienar, Liviu Pop, Marilena Lăzărescu, Radmila Costăchescu, Mirela Mogoi, Ruxandra Mare, Edward Șeclăman

**Affiliations:** 1 Department of Pediatrics, 2nd Pediatrics Clinic, “Victor Babes” University of Medicine and Pharmacy, 300041 Timisoara, Romania; pienar.corina@umft.ro (C.P.);; 2 The Necker-Enfants Malades Hospital, University of Paris Descartes, 75006 Paris, France; 3 Gastroenterology Department, “Victor Babes” University of Medicine and Pharmacy, 300041 Timisoara, Romania; 4 Biochemistry Department, “Victor Babes” University of Medicine and Pharmacy, 300041 Timisoara, Romania

**Keywords:** lactose intolerance, polymorphisms, dairy intake, quality of life, children

## Abstract

Background: Primary lactose intolerance (PLI) is characterized by the inability to digest lactose. Homozygotes for the lactase gene polymorphisms (CC or GG) are considered to be genetically predisposed to PLI. Still, symptoms may only be present later in life. The evidence supporting a link between PLI, dairy intake, and quality of life (QoL) is limited in children. Aim: This study investigates the link between LCT polymorphisms and suggestive symptoms and the influence of the genetic predisposition to PLI on dairy intake and QoL in Romanian children. Materials and methods: We recruited consecutive children evaluated in our ambulatory clinic. We asked all participants to complete a visual-analog symptoms scale, a dairy intake, and a QoL questionnaire. We used strip genotyping to identify genetic predisposition to PLI. Results: 51.7% of children had a CC genotype, and 34.5% also had a GG genotype. Most children reported no or mild symptoms. Dairy intake and QoL were similar across study groups. Conclusions: Our study shows that genetic predisposition does not necessarily assume the presence of specific symptoms. Genetic predisposition to PLI did not lead to dairy avoidance, nor did it negatively influence our children’s QoL.

## 1. Introduction

Primary lactose intolerance (PLI) is the most common form of disaccharide deficiency, characterized by the inability to digest lactose secondary to a low level of the enzyme lactase [1]. Lactase is located at the surface of the intestinal mucosa and has a maximum level immediately after birth, decreasing progressively with age [2]. Thus, PLI is caused by a genetically programmed reduction in lactase production after childhood, beginning at age 6.

The lactase gene (LCT) has 50 kilobases and is located on the long arm of chromosome 2 [3]. Since the absence of lactase from the mucosal surface and, subsequently, lactose intolerance is anthropologically “normal”, the persistence of lactase and lactose tolerance has developed through mutations [4]. C/T13910 and G/A22018 are the polymorphisms responsible for lactase persistency and lactose tolerance. Located in the promoter region of the LCT gene, C/T13910 and G/A22018 consist of upstream and downstream substitutions of 14 and 22 kilobases of the 5 ‘end of the lactase gene [5].

C/T13910 polymorphism seems dominant, while allele C is responsible for decreased enzyme expression [3,5]. Although the G/A22018 is not as common, allele G leads to similarly low lactase production. As adults, homozygous (CC and GG, respectively) individuals are left with undetectable lactase levels, while heterozygosity leads to moderately low enzyme activity in the mucosal surface of the small bowel. Both TT and AA genotypes correlate with lactase persistency and lactose tolerance [3,5].

Lactase activity begins to decrease around the age of 2 years. Still, the earliest symptoms of PLI appear after age 6 [5]. These include abdominal pain, borborygmi, flatulence, diarrhea, nausea, and vomiting [1].

Per current guidelines [6], PLI diagnosis is made via the hydrogen breath test. If the child cannot perform the hydrogen breath test, other tests, such as molecular testing, assessment of lactase activity in intestinal biopsies, and lactose or gaxylose test, are employed. Still, they are not routinely recommended in diagnosing PLI [6].

Although HBT is easy to perform, non-invasive, cheap, highly sensitive, and specific [7], it has some significant disadvantages, especially for the pediatric population. The HBT consists of 12 to 24 measurements, one every 15 min, for 3 to 6 h periods, and there can be a high number of false-negative or false-positive tests in everyday pediatric situations (e.g., physical exercise, recent use of oral antibiotics, use of probiotics, SIBO, constipation). Some might argue the need for standardization criteria of the indications, methodology, and interpretation of the results [8,9].

In order to overcome these issues, genetic testing became an attractive alternative. There is evidence that genetic testing for LCT polymorphisms in adults and children correlates very well with positive hydrogen breath tests [10,11,12,13,14,15]. Furthermore, compared with the assessment of lactase activity in intestinal biopsies, the identification of LCT gene polymorphisms showed excellent specificity and sensitivity for detecting PLI in children above 12 [16]. As such, some authors suggest molecular testing as a replacement for hydrogen breath tests [12,13]. However, even though the genetic predisposition for PLI, defined as the presence of LCT polymorphisms, is present from birth, actual lactose intolerance symptoms may not be present until young adulthood [15,17].

Although milk and dairy products are very often suspected by individuals, parents, and medical professionals to cause many digestive symptoms, their dietary exclusion may lead to nutrient imbalances. Thus, without proper diagnosis and subsequent nutritional management, a dairy-exclusion diet may have a negative impact on health and growth [1,5,18]. Furthermore, medically unsupervised restrictive diets can result in food aversions and eating disorders [19,20].

There is evidence that lactose intolerance, genuine or perceived, may negatively influence milk and dairy product intake., A low milk and dairy intake may predispose adults to poor bone health, osteoporosis, and fractures [18]. Furthermore, several studies have linked the dominant LCT polymorphism to low dairy intake in adults [21,22,23,24].

In children, on the other hand, the evidence supporting a link between PLI and dairy intake is scarce and conflicting. As such, some authors did not find an association between the dominant LCT polymorphism and dairy intake [15]. Others showed the long-term necessity of milk and dairy avoidance [25].

Although not a life-threatening disease, PLI may leave its mark on an individual’s quality of life [20]. Studies show that adults with PLI have an increased rate of digestive symptoms and lower HRQoL [24,26,27,28]. Suggesting a „toxin” theory, some authors describe a direct negative mechanism between lactose intolerance and quality of life [29]. In contrast, there is evidence that lactose-intolerance-suggestive symptoms are significantly associated with higher somatization indexes [30]. Still, somatoform disorders are associated with depression and anxiety, leading to an even poorer HRQoL.

Currently, there are limited data regarding the impact of lactose intolerance on HRQoL in children. Studies suggest that lactose intolerance does not impact the quality of life of children with digestive symptoms [25,31].

This study aims to investigate the link between LCT polymorphisms and lactose-intolerance-suggestive symptoms, as well as the influence of the genetic predisposition to PLI on dairy intake and health-related quality of life (HRQoL) in Romanian children.

## 2. Materials and Methods

### 2.1. Study Design

We conducted a cross-sectional study, recruiting consecutive children who needed blood drawn in our ambulatory clinic over a period of four months: May–August 2016. We enrolled children between 6 and 18 years of age at the time of inclusion in the study. We excluded children with any acute illness, children with malabsorption syndromes (including celiac disease, cow’s milk protein allergy, secondary lactose intolerance, cystic fibrosis, and IBD), children born premature, LGA/SGA, children with diabetes mellitus, metabolic syndrome, obesity, children on medication that influences blood pressure, blood glucose and lipids, and children or parents who refused to take part in the study. Our study population consisted of 87 children.

### 2.2. Analyzed Variables

**Figure A1:**
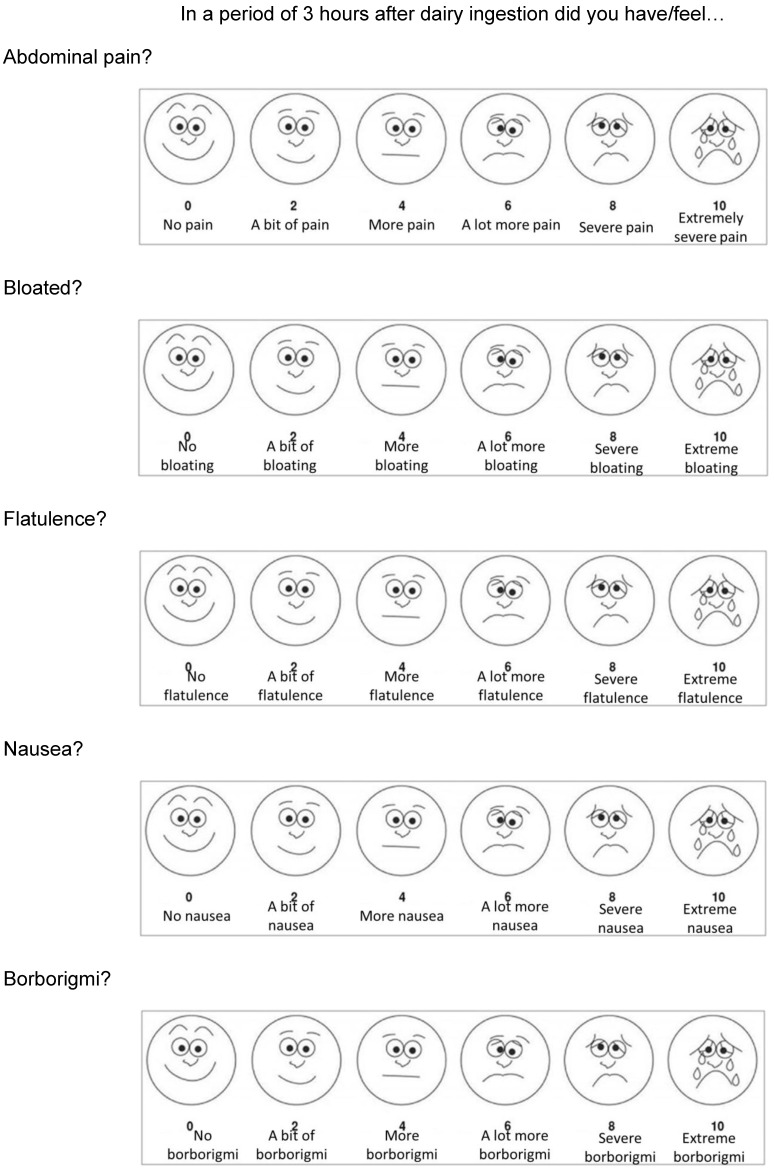
Visual Analog Scale: lactose-intolerance-related symptoms.

We measured weight (kg) and height (cm) in all children and calculated the body mass index (BMI, weight/square height, kg/m^2^). We asked children or parents to complete an analog visual scale for digestive symptoms associated with PLI (Appendix A, Figure A1). The scale consisted of the following PLI-related symptoms: abdominal pain, abdominal distension, flatulence, nausea, and borborygmi. Each symptom was scored: 1 point per symptom. For the analysis, we considered children with scores from 0 to 2 to have absent/mild symptoms. We considered children with scores between 4 and 10 to have moderate/severe symptoms.

**Figure A2:**
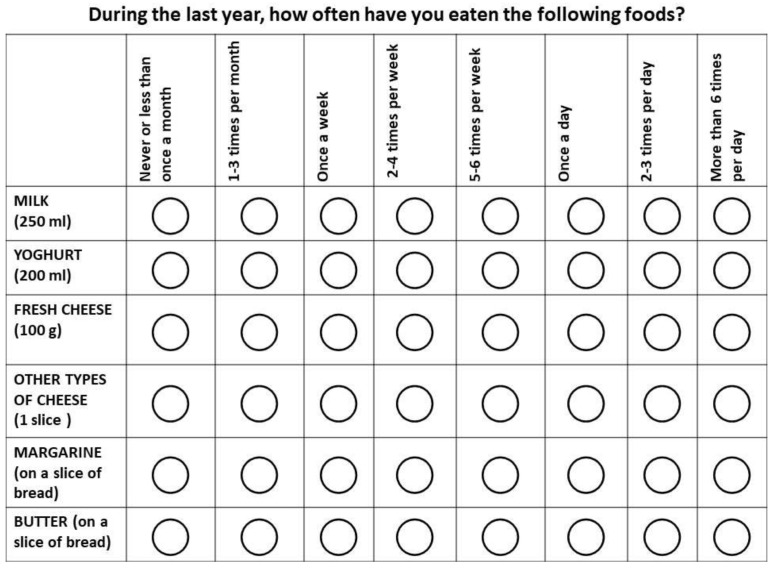
Dairy intake questionnaire.

Parents also had to take a dairy intake questionnaire, as presented in Appendix A, Figure A2. The form included the essential types of dairy: milk, yogurt, cheese, butter, and margarine. The form included 8 points of dairy intake, ranging from “never/less than once a month” to “more than 6 times per day”. For the analysis, the first four points in the dairy intake questionnaire assumed a low dairy intake, while the last four points assumed a high dairy intake.

**Figure A3:**
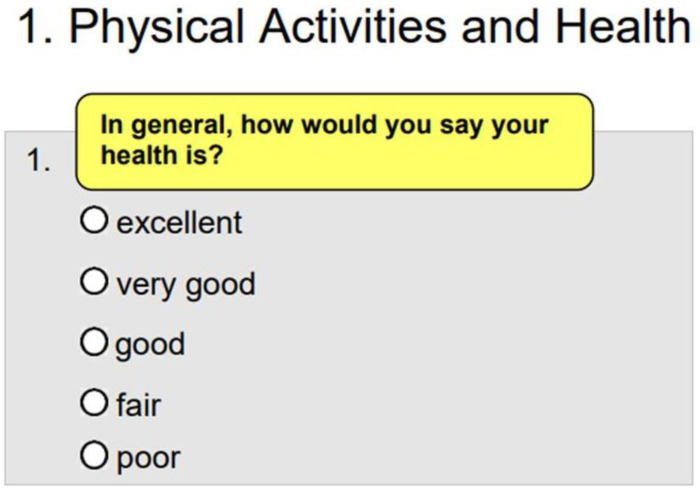
Validated QoL questionnaire (Kidscreen).

We asked children over 8 years of age or parents to complete a validated quality-of-life questionnaire (Kidscreen, Appendix A, Figure A3). While we used a complete quality-of-life questionnaire, we focused in this paper on the HRQoL, namely the first question of the validated questionnaire. When asked, “How do you think your health is?” the children or parents could choose between “excellent”, “very good”, “good”, “fair”, or “poor”.

### 2.3. Molecular Analysis

In order to identify genetic predisposition for PLI, we used strip genotyping, a molecular diagnosis method certified for in vitro diagnosis. Thus, we obtained rapid and reliable results. This technique has very high specificity and sensitivity and consists of an amplification reaction followed by reverse hybridization. For this analysis, a drop of whole blood was collected and stored on a special support, GenoCard (Hain Lifescience GmbH, Nehren, Germany). Homozygous children for at least one polymorphism were lactose intolerant (*n* = 42), while the others were lactose tolerant (*n* = 45). As a result, we considered children with a genetic predisposition to PLI lactose intolerant (LiT), while considering children without the genetic predisposition were considered lactose tolerant (LT).

### 2.4. Statistical Analysis

According to the variable type (ordinal or continuous), we expressed our results as frequencies and mean ± standard deviation. The t, Mann–Whitney, and Kruskal–Wallis tests (based on the distribution of the analyzed variables) were used to assess the differences in gender, anthropometrics, symptoms, dairy intake, and HRQoL between LiT and LT children. We used a confidence interval of 95% for all tests we performed. The statistical package IBM SPSS Statistics 17 (IBM Company, Armonk, NY, USA) was used for the statistical analysis.

### 2.5. Ethics

We conducted our study following the principles of ethics described in the Helsinki Declaration. The study has been approved by the “Victor Babes” University of Medicine and Pharmacy Ethics Committee (18/2016). Parents or legal guardians and, where appropriate, children gave informed consent to participate in the study.

## 3. Results

### 3.1. Genetic Predisposition to PLI and PLI-Related Symptoms

Gender and mean age, weight, height, and BMI were similar in the two studied groups (Table 1).

When looking at the specific genotypes, homozygosity for one or both polymorphisms and heterozygosity or absence of the polymorphisms, gender and mean age, weight, height, and BMI remained similar (Table 2).

**Figure 1:**
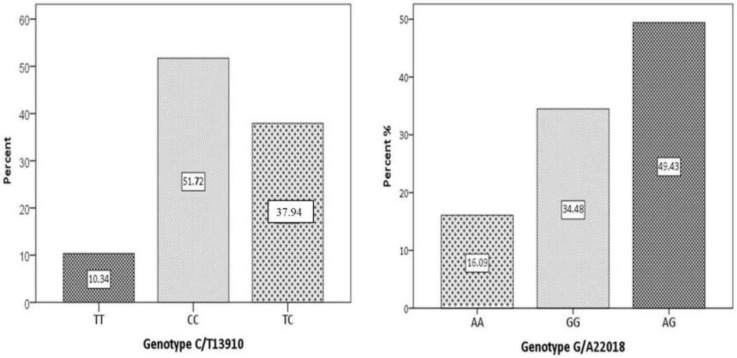
The distribution of LCT polymorphisms in the studied population: left-C/T13910 (dominant genotype); right-G/A22018. CC/GG denotes homozygosity for the respective polymorphism; TT/AA denotes the absence of the respective polymorphism; TC/AG denotes heterozygosity of the respective polymorphism.

As seen in Figure 1, 45 (51.72%) children had a CC genotype. Thus, these children had a genetic predisposition to PLI. Of these, 30 children also had a GG genotype (Figure 1). Forty-two children had either heterozygosity or absence for the two polymorphisms. Thus, they did not have a genetic predisposition for PLI. Our results show consistency with the Hardy–Weinberg equilibrium.

**Figure 2:**
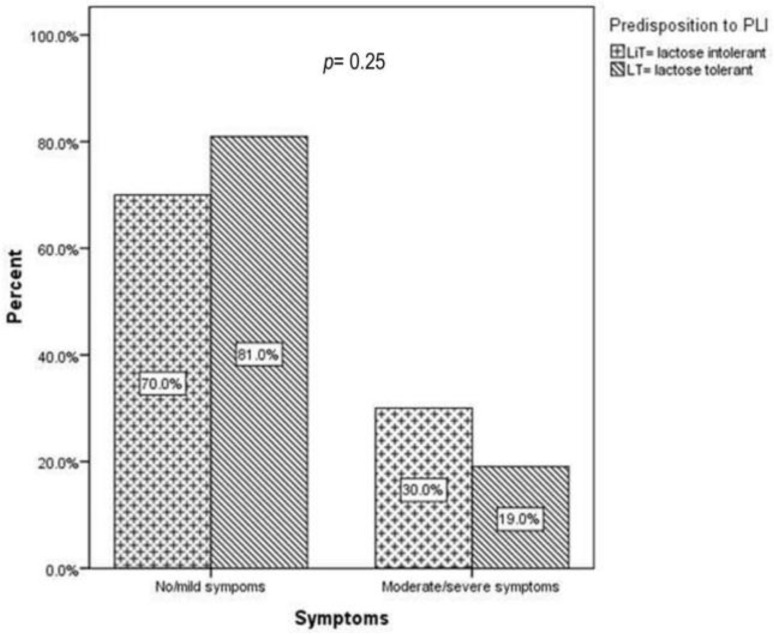
Primary lactose-intolerance-suggestive symptoms across study groups: children with a genetic predisposition to primary lactose intolerance (LiT) versus children without genetic predisposition (LT); PLI denotes primary lactose intolerance; *p* represents the statistical significance of the Mann–Whitney test.

Most children reported no or mild PLI-related symptoms (70% vs. 81%), as presented in Figure 2. Lactose-intolerance-suggestive symptoms follow a similar distribution in children with a genetic predisposition to PLI (LiT) compared to children without a genetic predisposition (LT). Although not statistically significant, 14/22 children from the group with moderate/severe symptoms had a genetic predisposition to PLI.

**Figure 3:**
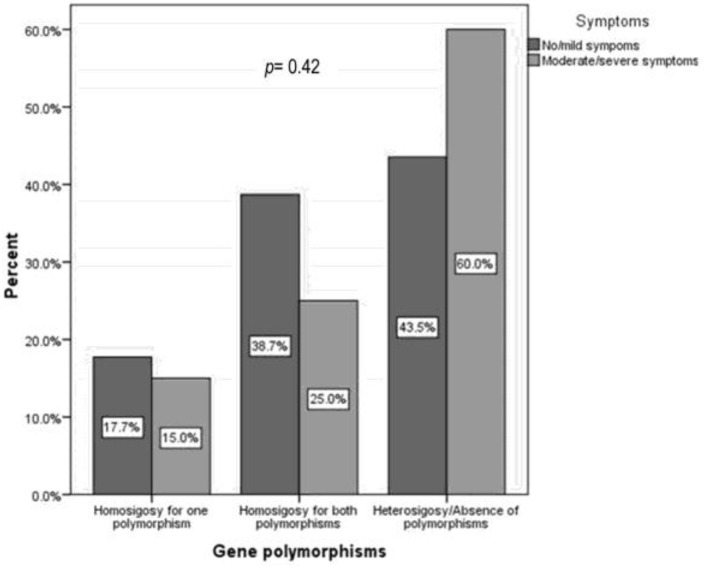
Primary lactose-intolerance-suggestive symptoms according to genotype: homozygosity for one or both polymorphisms and heterozygosity or absence of the polymorphisms. *p* represents the statistical significance of the Kruskal–Wallis test.

Furthermore, as shown in Figure 3, the presence or one or both of the polymorphisms did not influence the severity of symptoms distribution (17.7% vs. 15% and 38.7% vs. 25%, *p* = 0.42).

### 3.2. Genetic Predisposition to PLI and Dairy Intake

**Figure 4:**
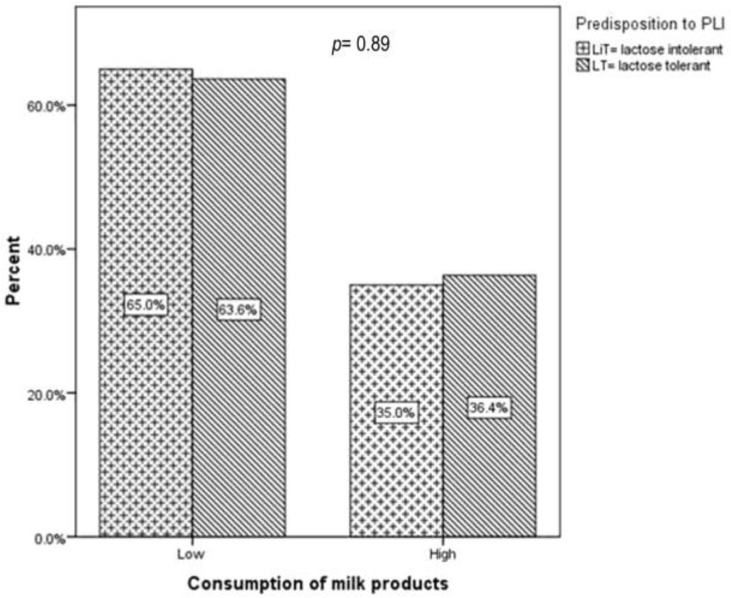
Dairy intake across study groups: children with a genetic predisposition to primary lactose intolerance (LiT) versus children without a genetic predisposition (LT); PLI denotes primary lactose intolerance; LiT, lactose intolerant; LT, lactose tolerant. *p* represents the statistical significance of the Mann–Whitney test.

Figure 4 shows that we did not find significant differences in dairy intake in children with a genetic predisposition for PLI (LiT) compared to children without this predisposition (LT).

**Figure 5:**
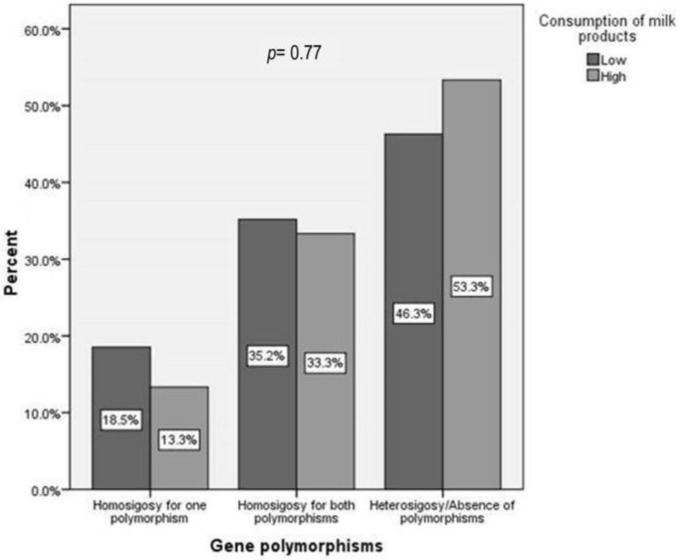
Dairy intake according to genotype: homozygosity for one or both polymorphisms and heterozygosity or absence of the polymorphisms. *p* represents the statistical significance of the Kruskal–Wallis test.

In addition, milk consumption distribution was similar in children with one or both polymorphisms: 18.5% vs. 13.3% and 35.2 vs. 33.3, *p* = 0.77 (Figure 5).

The fact that two-thirds of children declare a low dairy consumption (65% vs. 63.6%) is nevertheless worrisome.

### 3.3. Genetic Predisposition to PLI and HRQoL

**Figure 6:**
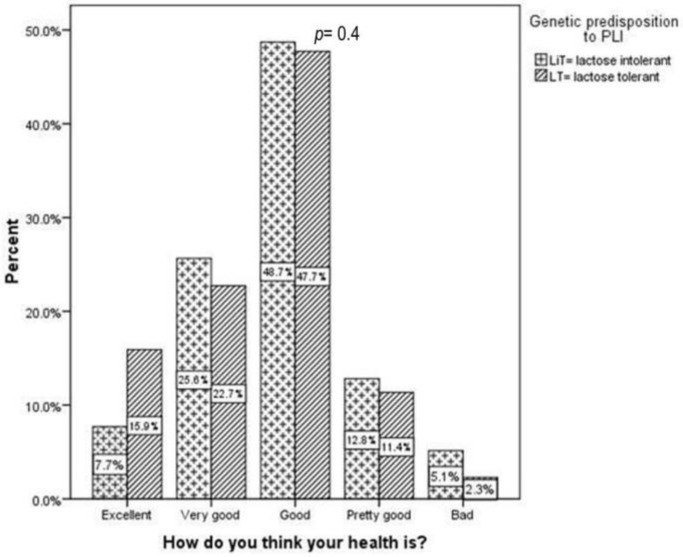
HRQoL across study groups: children with genetic predisposition to primary lactose intolerance (LiT) versus children without genetic predisposition (LT); HRQoL denotes health-related quality of life; LiT, lactose intolerant; LT, lactose tolerant. *p* represents the statistical significance of the Mann–Whitney test.

As shown in Figure 6, HRQoL was similar in children with a genetic predisposition to PLI (LiT) and those without this predisposition (LT).

**Figure 7:**
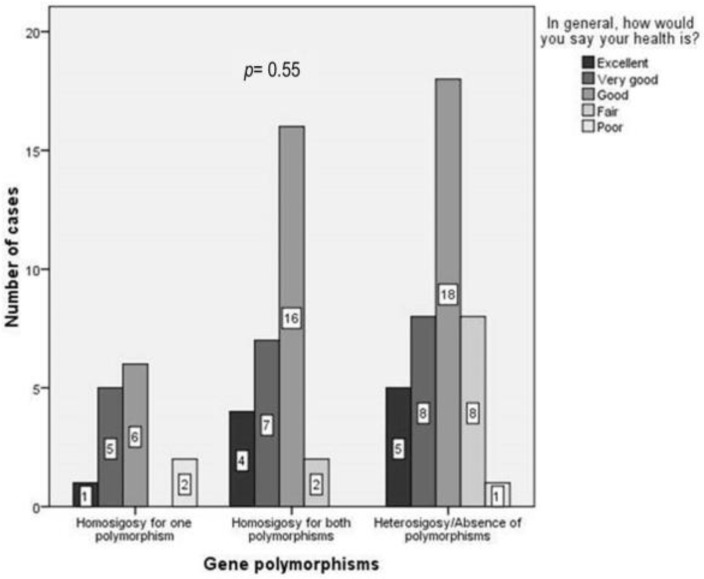
HRQoL according to genotype: homozygosity for one and both polymorphisms and heterozygosity or absence of the polymorphisms.; HRQoL denotes the health-related quality of life. *p* represents the statistical significance of the Kruskal–Wallis test.

Children with homozygosity for both polymorphisms reported HRQoL similar to their peers (Figure 7).

When legal guardians or children were asked how they perceived their health (Figure 6), the vast majority responded that they believed their health was “excellent” (7.7% vs. 15.9%), “very good” (25.6% vs. 22.7%), or “good” (48.7% vs. 47.7%). Just a small percentage of parents or children perceived their health as being “fair” (12.8% vs. 11.4%) or “poor” (5.3% vs. 2.3%).

## 4. Discussion

### 4.1. Genetic Predisposition to PLI and PLI-Related Symptoms

Worldwide, almost two-thirds of humans lose their ability to digest lactose after the weaning age. The persistence of the enzyme lactase at the „brush” border of the intestinal mucosa is most frequent in populations with a long history of consuming milk and dairy products [32]. The development of LCT gene polymorphisms that determine lactase persistence mirrors the history of milk consumption across different geographical areas [3,32]. Thus, it is evident that lactose intolerance is most prevalent in Arabic, Jewish, East Asian, and West African people [33,34]. In Europe, the prevalence of LCT polymorphisms is rising from north to south. As such, only about 5% of Northern Europeans lose their ability to digest lactose in adulthood [3]. The trend of lactose intolerance prevalence increases to about 30% in Central Europe, and rises to more than 70% in Southern Europe. As we previously showed [35], our study was the first to evaluate PLI in the Romanian pediatric population, founding a PLI frequency of 51.7%. These findings are consistent with the Hardy–Weinberg equilibrium and overlap with European PLI prevalence. In our study, 30/45 children were homozygous for both CC and GG genotypes. This finding was also observed by Tomczonek-Moruś et al., who reported that patients with homozygous 22018-GG also have 13910-CC, but not vice versa [14]. More than ten years ago, studies showed that in specific adult populations, the 22018 G>A polymorphism is a better indicator of PLI than the 13910 C>T [36,37]. Still, our results do not show an increase in symptoms’ severity in children with homozygosity for both polymorphisms.

Lactose intolerance must be suspected in the context of non-specific digestive symptoms (e.g., nausea, abdominal pain and meteorism, borborygmi, and loose stools) that appear a few hours after milk and dairy intake. They represent an individual’s genetically programmed inability to digest lactose after the weaning age.

There are considerable intra- and inter-individual variations in the severity of PLI symptoms, depending on the quantity of ingested lactose and the individual’s ability to digest it. These variations are determined by numerous factors, including osmolality and lipid content of foods that contain lactose, gastric emptying time, lactose digestion capacity, the speed of gastrointestinal transit, the water absorption capacity of the colon, and the perception of abdominal pain and discomfort of each individual [3,34,38,39]. In addition, symptoms of functional gastrointestinal disorders (FGIDs) as defined by Rome IV criteria are similar and overlap with lactose-intolerance-suggestive symptoms [40]. Furthermore, current guidelines do not recommend using the HBT in diagnosing children with abdominal-pain-related FGIDs [6]. On the other hand, there is evidence that a lactose-free diet might alleviate FGID symptoms [19,24]. Thus, the causal relationship between lactose intake and digestive symptoms is complicated.

In our study, most children or parents reported no or mild symptoms (*n* = 65/87). Furthermore, we found no significant differences in the severity of digestive symptoms in children with LCT polymorphisms compared to their peers with ancestral variants. In addition, in the small group of children reporting moderate-to-severe digestive symptoms, just two-thirds had a genetic predisposition to PLI. As suggested by Kerber M et al., Di Stefano M et al., and Couce ML et al. [11,15,17], the high number of children with a genetic predisposition to PLI presenting with no/mild symptoms might be explained by the appearance of suggestive symptoms later in life [11,15,17]. This finding could also be attributed to each individual’s variability in digesting lactose. This hypothesis was tested by Nicklas et al. in a nationally representative African American and Hispanic adult population. They reported that only 12–13% had PLI-suggestive symptoms, although the overall prevalence of genetic predisposition to PLI was higher [41].

In contrast, Gremse et al. evaluated symptoms associated with milk intake in European children with PLI. 30 children (11 boys) aged 3 to 17 were included. After ingesting 240 mL of milk daily for 14 days, abdominal pain significantly increased after lactose ingestion compared to the lactose-free period [42]. In addition, Yerushalmy-Feler A showed that of 203 children with digestive symptoms tested via HBT, 154 had positive results. Furthermore, almost half of the children (42.2%) experienced a clinical relapse when dairy was reintroduced. [25].

Our study shows that PLI does not explain moderate/severe symptoms in children with LCT polymorphisms (lactose tolerant). Since we excluded children with organic causes of digestive symptoms, FGIDs could explain the complaints and allow for different therapeutic approaches. Almazar AE et al. have also shown that lactose-intolerance-suggestive symptoms in adults with irritable bowel syndrome are self-reported with high frequency but correlate poorly with LCT polymorphism genotype [10].

### 4.2. Genetic Predisposition to PLI and Dairy Intake

In children, long-term dietary exclusion of milk products is associated with lower height and bone mineral density, and higher fracture risk [33,43]. In our study, dairy intake was not higher in lactose-tolerant children than in children with genetic predisposition PLI. This finding was also reported by Casellas et al. They showed that objective PLI in adults was not clearly associated with avoiding dairy products [44]. On the other hand, several studies have shown dairy avoidance behavior in adults with PLI genotypes [21,22,23,24]. In addition, Obermayer-Pietsch et al. found that dairy intake was significantly lower in women with PLI [45]. Furthermore, an analysis of musculoskeletal diseases performed by Schiffner et al. showed that patients with PLI had osteoporosis more frequently than healthy individuals [46].

In children, however, in accordance with our findings, a recent study that included 493 children, Couce ML et al. did not show a link between PLI genotype and milk and dairy consumption [15]. In contrast, Yerushalmy-Feler A et al. showed that children diagnosed with PLI through HBT were still on a dairy-free diet more than three years after diagnosis [25].

Nevertheless, when Kull et al. studied the diary intake of 367 Estonian adults aged 25–70, they found that self-perceived PLI leads to self-imposed diary intake reductions. Furthermore, they proved that self-perception of PLI, rather than affection itself, influenced dairy intake [47]. Thus, genetic predisposition to PLI might lead to dairy avoidance in symptomatic adults and older children. Still, the patient’s perception of the intolerance carries much weight when deciding to avoid milk products.

Similarly to what Couce ML et al. observed in their cohort of pediatric patients (61%) [15], more than half of the children in our study (64.3%) reported low dairy consumption. This high percentage of low dairy intake could be influencing our results. It is known that most individuals with lactose intolerance can tolerate moderate milk consumption without experiencing moderate or severe digestive symptoms [8,33,48,49].

### 4.3. Genetic Predisposition to PLI and HRQoL

Lactose intolerance impacts a person’s quality of life on different levels: at an individual level by presenting with bothersome digestive symptoms, and at a social level, it is associated with high psycho-social stress levels which lead to anxiety disorders and FGIDs [20].

Our study did not find a lower HRQoL in children with a genetic predisposition for PLI than in lactose-tolerant children. Furthermore, children homozygous for both polymorphisms reported similar HRQoL to children homozygous for just one polymorphism. In accordance with our results, Yerushalmy-Feler et al. found an overall good quality of life in 203 children with lactose-intolerance-suggestive symptoms, irrespective of the results of their hydrogen breath tests [25]. Similarly, Strinnholm et al. reported a good quality of life in 33 Swedish children with PLI [31].

On the other hand, adult data suggest a link between PLI and anxiety concerning milk and dairy intake, which may lead to an impaired HRQoL [20]. This difference between children and adults with PLI may be explained by the results of Casellas et al., Jansson-Knodell et al., and Zheng et al., who all found that self-perceived lactose intolerance in adults was associated with lower scores on the quality-of-life questionnaires [24,28,44]. However, HRQoL scores were significantly lower in those with PLI than in healthy subjects [44]. Once again, individual self-perception of lactose intolerance heavily influences one’s HRQoL.

### 4.4. Limitations of the Study

The most significant limitation of our study is the small number of enrolled children.

We acknowledge that an underpowered study may lead to a type II statistical error (believing there are no differences between study groups, when, in fact, there are). Additionally, our population included children who visited our outpatient clinic, making it a convenience sample.

Still, the molecular testing results are as expected when looking at the PLI gradient across Europe. In addition, our findings show consistency with the Hardy–Weinberg equilibrium. Still, further studies are needed to verify our results.

## 5. Conclusions

Our study shows that genetic predisposition to PLI does not necessarily assume the presence of lactose-intolerance-related symptoms in a pediatric population with a moderately high PLI frequency. Other etiologies, including FGIDs, need to be explored even in the presence of suggestive symptoms.

Genetic predisposition to PLI did not lead to dairy avoidance, nor did it negatively influence our children’s HRQoL. In an age when genetic testing is readily available with or without proper indication, our results emphasize that pediatric genetic predisposition to PLI alone, without appropriate clinical context or HBT confirmation, cannot justify a milk- and dairy-exclusion diet.

Thus, a restrictive diet might not be necessary or beneficial for all children. Our goal must be to promote a balanced and unnecessarily restricted diet throughout childhood and adolescence.

However, due to the limited study population and variability of the methodology employed in the comparison studies, the dairy intake and HRQoL results need careful interpretation. Thus, our results need to be tested by larger and adequately powered studies.

## Figures and Tables

**Table 1:** Characteristics of the study groups, according to the presence or absence of the genetic predisposition for primary lactose intolerance.

		Lactose Tolerant *n* = 45	Lactose Intolerant *n* = 42	*p* ^1^
Age (years) ^2^		11.09 ± 3.59	10.17 ± 3.4	0.221
Gender ^3^	Girls	21/45	24/42	0.744
	Boys	24/45	18/42	
Weight (kg) ^2^		42.92 ± 16.94	39.07 ± 17.41	0.372
Height (cm) ^2^		144.37 ± 18.38	148.67 ± 18.06	0.273
BMI (kg/mp) ^2^		19 ± 5.08	17.99 ± 4.65	0.440

^1^ *p* represents the statistical significance of the *t*-test. BMI denotes body mass index. ^2^ Data are expressed as means ± standard deviations. ^3^ Data are expressed as number of girls/boys in each group.

**Table 2:** Characteristics of the study population, according to the genotypes.

		Homozygosy for Genotype 13910 (*n* = 15)	Homozygosy for Both Genotype 13910 and 22018 (*n* = 30)	Heterozygosy/Absence of Polymorphisms (*n* = 42)	*p* ^1^
Gender ^2^	Girls	7	21	17	0.045
	Boys	8	9	25	
Age (years) ^3^		9.71 ± 3.14	11.2 ± 3.52	10.62 ± 3.64	0.406
Weight (kg) ^3^		39.89 ± 17.79	43.73 ± 17.54	38.24 ± 15.57	0.355
Height (m) ^3^		142 ± 18.69	148 ± 19.03	145.13 ± 18.59	0.549
BMI (kg/m^2^) ^3^		18.86 ± 5.38	19.19 ± 4.67	17.87 ± 4.67	0.362

^1^ *p* represents the statistical significance of the Kruskal–Wallis test. BMI denotes body mass index. ^2^ Data are expressed as number of girls/boys in each group. ^3^ Data are expressed as means ± standard deviations.

## Data Availability

The datasets used and/or analyzed during the current study are available from the corresponding author on reasonable request.
